# Transcriptome Analysis Reveals *PpMYB1* and *PpbHLH1* Promote Anthocyanin Accumulation in *Phalaenopsis pulcherrima* Flowers

**DOI:** 10.3390/biom15070906

**Published:** 2025-06-20

**Authors:** Jianqiang Wen, Ji Li, Kunlin Wu, Jingjue Zeng, Lin Li, Lin Fang, Songjun Zeng

**Affiliations:** 1 Key Laboratory of South China Agricultural Plant Molecular Analysis and Gene Improvement, South China Botanical Garden, Chinese Academy of Sciences, Guangzhou 510650, China; wenjq@scbg.ac.cn (J.W.); liji17@scbg.ac.cn (J.L.); wkl8@scib.ac.cn (K.W.); zengjingjue@scbg.ac.cn (J.Z.); lilin@scbg.ac.cn (L.L.); 2 Guangdong Provincial Key Laboratory of Applied Botany, South China Botanical Garden, Chinese Academy of Sciences, Guangzhou 510650, China

**Keywords:** flower color, RNA-seq, transcription factors, MYB, bHLH

## Abstract

*Phalaenopsis pulcherrima* are known for their captivating floral morphology and diverse colors, demonstrate exceptional resilience to adverse environmental conditions, and exhibit significant potential for hybrid breeding. However, current research on flower coloration is still limited. The data from this study indicates that variations in anthocyanin levels are the primary determinants of the difference between white and purple colors. Through RNA-seq, we identified 469 genes that were differentially expressed. Furthermore, our bioinformatics exploration uncovered two potential transcription factors, PpMYB1 and PpbHLH1, which play regulatory roles in anthocyanin accumulation. Y2H assays demonstrated that these two TFs could form heterodimers and interact with each other. Afterwards, transient expression assays were conducted for the first time in *P. pulcherrima* flowers, revealing that overexpression of *PpMYB1* alone or in combination with *PpbHLH1* resulted in purple petal pigmentation. Overexpressing *PpMYB1* in tobacco resulted in more purple-colored corollas, stamens, pistils, and pods compared to control plants. Y1H and dual-luciferase assays provided further evidence that PpMYB1 and PpbHLH1 interact with the promoters of the structural genes *PpF3H*, *PpDFR*, and *PpANS* in the anthocyanin biosynthesis pathway, thereby driving their robust expression. This study not only enhances our understanding of the molecular mechanisms underlying anthocyanin synthesis but also holds significant practical implications for advancing plant hybrid breeding and genetic engineering applications in flower color regulation.

## 1. Introduction

*Phalaenopsis pulcherrima* (Lindl.) J. J. Sm., also known as *Doritis pulcherrima*, was historically grouped under the *Doritis* genus but has since been reassigned to the *Phalaenopsis* genus in the Orchidaceae family [1,2]. It is an endemic species in East Asia, with its native habitats in Hainan, China, and Southeast Asia. The biggest difference between *P. pulcherrima* and *Phalaenopsis* is that the labellum of *P. pulcherrima* has five lobes, while the labellum of *Phalaenopsis* has only three lobes. *P. pulcherrima* has a beautiful flower shape, with different colors for the sepals, petals, and lips. The flower colors include pink, purple, white, orange, etc. It has strong stress resistance, especially with a flowering period in summer and autumn, which is different from most *Phalaenopsis* species that bloom in spring. It has high ornamental value and application prospects and is an important parent for the hybrid breeding of *Phalaenopsis* [3,4].

Anthocyanin is one of the most critical factors affecting the flower color of ornamental plants, and it also plays an important role in the growth and development of plants [5]. It can protect plants from strong light, ultraviolet rays, and low temperatures [6] and improve the plant’s resistance [7]. The biosynthetic pathway of anthocyanins is the most extensively studied secondary metabolic pathway in plants [8,9]. The synthesis of anthocyanins within cells primarily occurs in the cytoplasm surrounding the vacuoles, through the phenylpropanoid pathway and the flavonoid biosynthetic pathway [10]. The biosynthesis of anthocyanin is mainly controlled by two types of genes, namely structural genes and regulatory genes [11]. Structural genes are responsible for coding enzymes that catalyze the biochemical reactions involved in anthocyanin biosynthesis. These genes can be categorized into two major groups: the Early Biosynthetic Genes (EBGs) and the Late Biosynthetic Genes (LBGs) [12].

EBGs primarily regulate the production of flavanonol precursors, such as Chalcone Synthase (CHS), Chalcone Isomerase (CHI), Flavanone 3-Hydroxylase (F3H), Flavonoid 3′-Hydroxylase (F3′H), and Flavonoid-3′,5′-Hydroxylase (F3′5′H) [13]. CHS, as the initial enzyme in this process, catalyzes the first step of anthocyanin biosynthesis [14]. Its inactivity results in the loss of flavonoids, including anthocyanins, flavones, and flavonols, leading to the development of white mutants [15]. By suppressing the activity of CHI, which serves as the second key enzyme, yellow flowers can be obtained through the accumulation of yellow chalcones and chalcone derivatives [16]. F3H catalyzes the formation of the colorless dihydrokaempferol, serves as a key regulatory point in the pathway, and is the last enzyme in the EBGs of the flavonoid synthesis pathway, and its expression level directly influences the development of red pigmentation. When arginine at position 130 of the red strawberry *F3H* gene mutates to histidine, it results in a pink phenotype [17]. Similarly, antisense suppression of F3H expression in other plants can lead to a reduction or complete loss of their original orange or reddish flower coloration [18]. In contrast, the LBG family is essential for the production of various anthocyanidins, which serve as vital intermediates in the determination of anthocyanin pigmentation. These genes are typically modulated by transcriptional regulators [19,20]. Enzymes within this family include Dihydroflavonol 4-Reductase (DFR), Anthocyanidin Synthase (ANS), and UDP-Flavonoid Glucosyl Transferase (UFGT), all of which are integral to the biosynthetic pathway [21,22].

Regulatory genes primarily encompass three key transcription factor families: MYB, bHLH, and WD40 [23]. MYB and bHLH proteins are capable of independently influencing anthocyanin biosynthesis, while they can also interact with WD40 proteins to form a MYB-bHLH-WD40 complex, which specifically targets the promoters of structural genes [24]. This complex is essential for either suppressing or stimulating the expression of these genes, thereby controlling the synthesis and spatial distribution of anthocyanins [25]. The initial MYB transcription factor identified in plants was *ZmMYBC1*, which was isolated from maize in 1987 [26]. Following this discovery, scientists have employed advanced molecular techniques, such as functional genomics, transcriptomics, and proteomics, to identify and characterize numerous R2R3-MYB transcription factors that actively promote anthocyanin synthesis in various plant species such as *Michelia maudiae* [27], *Hibiscus syriacus* L. [28], cotton [29], grapes [30], and *Cymbidium lowianum* [31]. In the model organism *Arabidopsis*, the TTG1 (WD repeat protein) interacts with TT2 (MYB) and TT8 (bHLH) to form a robust tripartite complex that induces the transcription of *BANYULS* (*BAN*), the gene encoding the central enzyme in proanthocyanidin synthesis pathways [32]. Recent studies have shown that the MYB transcription factor *AcMYB1* and the bHLH protein *AcbHLH1* significantly enhance anthocyanin levels in *Aglaonema commutatum* under light conditions [33]. In *Freesia hybrida*, co-transfection experiments using protoplasts indicate that *FhMYB5*, when expressed alongside bHLH genes, leads to a notable upregulation of both upstream and downstream flavonoid biosynthetic pathways [34]. Transcriptomic analysis of *Hippeastrum* × *hybridum* ‘Royal Velvet’ has identified a putative R2R3-MYB gene, *HpMYB1*, which plays a critical role in anthocyanin biosynthesis regulation [35]. Additionally, in *Phalaenopsis equestris*, the MYB transcription factor *PeMYB2* has been shown to specifically activate the expression of *PeF3H5*, *PeDFR1*, and *PeANS3*, all of which are key structural genes in the anthocyanin pathway [36]. Similarly, in *Oncidium* Gower Ramsay, the MYB protein OgMYB1 regulates the transcription of *CHI* and *DFR* genes, thereby facilitating anthocyanin production [37]. Additionally, the study also revealed R2R3-MYB transcription factors that can inhibit anthocyanin biosynthesis, such as *PhMYB27* [38], *TgMYB4* [39], *NtMYB2* [40], and *FaMYB1* [41]. The overexpression of these factors has been shown to decrease anthocyanin production.

In recent years, investigations into MYB transcription factors in orchids have expanded; nonetheless, no research has yet been reported on MYB transcription factors involved in anthocyanin biosynthesis within *P. pulcherrimum*. This study characterized one R2R3-MYB transcription factor (*PpMYB1*) and one bHLH transcription factor (*PpbHLH1*) and investigated their roles in anthocyanin biosynthesis in *P. pulcherrimum*. The findings demonstrated that PpMYB1 significantly elevates the expression levels of *PpF3H*, *PpDFR*, and *PpANS* genes associated with anthocyanin synthesis, both independently and in conjunction with *PpbHLH1*. Elucidating these transcription factors is crucial for understanding the regulatory mechanisms underlying anthocyanin biosynthesis in this species and holds potential for genetic engineering applications in other plants. Such insights will significantly contribute to the development of molecular breeding strategies aimed at improving the floral pigmentation of *P. pulcherrima* in the future.

## 2. Materials and Methods

### 2.1. Plant Materials and Growing Conditions

*P. pulcherrima* were introduced from Hainan and cultivated under controlled greenhouse conditions at the South China Botanical Garden, Chinese Academy of Sciences (Guangzhou, China). Four-year-old individuals of white- and purple-flowered varieties were selected as experimental materials. The plants were exposed to natural light filtered through a 60% shade cloth, under conditions of temperature ranging from 15–34 °C and relative humidity from 75–99%.

### 2.2. Determination of Anthocyanin Content

Anthocyanin levels were quantified following a modified protocol based on the method described by Wang et al. (2009) [42]. Samples of sepals, petals, and lips (approximately 0.05 g each) were powdered under liquid nitrogen conditions and subsequently poured into methanol supplemented with 90% (*v*/*v*) trifluoroacetic acid and reacted at 40 °C for 20 h. Centrifugation was performed at 10,000 rpm for 10 min using a microcentrifuge, and the supernatants were collected for analysis. Anthocyanin concentrations were determined via absorbance measurement at 519 nm using a Infinite 200 Pro microplate reader (Tecan, Männedorf, Switzerland). The anthocyanin content was calculated based on the following formula: Anthocyanin (μg/g) = (A519 × n × V)/(0.0772 × W), where A519 represents the absorbance at 519 nm, n denotes the dilution factor, V indicates the total volume of the extraction supernatant (mL), and W corresponds to the fresh weight of the tissue (g). Each tissue sample underwent three independent measurements, and data are presented as the mean ± Standard Error (SE).

### 2.3. RNA-Seq

Total RNA was extracted from 0.1 g of floral organ tissue using the E.Z.N.A.^®^ Plant RNA Kit (Omega Bio-tek, Inc. Norcr, GA, USA). The quality, concentration, and purity of the RNA were evaluated using an Agilent 2100 bioanalyzer (Agilent Technologies, San Jose, CA, USA) and the ND-2000 NanoDrop spectrophotometer (Thermo Scientific, Wilmington, DE, USA). High-quality RNA (1 µg) was utilized to prepare RNA-Seq libraries following the Illumina^®^ Stranded mRNA Prep protocol (San Diego, CA, USA). The sequencing of the cDNA libraries was conducted by Shanghai Majorbio Bio-pharm Biotechnology Co., Ltd. (Shanghai, China) using the Illumina HiSeq™ 6000 platform (Illumina, San Diego, CA, USA).

### 2.4. Differentially Expressed Gene (DEG) Analysis

To determine Differentially Expressed Genes (DEGs) between white and purple flower tissues, the expression levels of genes were quantified and normalized via the Transcripts per Million Reads (TPM) method using RSEM software (version 1.3.3). DESeq2 software (version 1.24.0) [43] was used to compare the differential expression levels between two samples, and a gene was deemed significantly differentially expressed if it exhibited a *p* adjust < 0.05 and a log2(fold change) ≥ 1. Subsequently, these DEGs were subjected to KEGG pathway enrichment analysis to explore their functional roles.

### 2.5. Quantitative Real-Time PCR Analysis

In this study, approximately 1 μg of total RNA was reverse-transcribed into cDNA using the StarScript III One-Step RT-PCR Kit (GenStar, Beijing, China). Quantitative Reverse Transcription Polymerase Chain Reaction (qRT-PCR) was conducted on a LightCycler 480 II system (Roche, Mannheim, Germany) with PerfectStart Green qPCR SuperMix from Transgen (Beijing, China). Transcript levels were normalized to the endogenous actin gene. Each sample is repeated three times. Relative gene expression was determined using the 2^−ΔΔCT^ method, as described by Livak [44]. Primers were designed with Primer 5.0, and their sequences are provided in Table S1.

### 2.6. Identification and Phylogenetic Analysis of Transcription Factors

The MYB and bHLH transcription factors were first subjected to sequence alignment using the default parameters of ClustalW and MUSCLE, both of which are integrated into MEGA version 5.2 [45]. A phylogenetic tree was then generated using the maximum likelihood method. To evaluate the confidence of the tree topology, 1000 bootstrap resampling replicates were performed. The sequences of R2R3-MYB and bHLH proteins were retrieved from the GenBank database (https://www.ncbi.nlm.nih.gov/), and the accession numbers of MYB and bHLH proteins are listed in Table S2.

### 2.7. Isolation of the Full-Length cDNA of PpMYB1 and PpbHLH1

The cDNA sequences of *PpMYB1* and *PpbHLH1* were successfully amplified through PCR. Specific primers were designed using Primer 5.0 (Table S1). The resulting PCR products were cloned into the pClone007 Versatile Simple Vector (Tsingke, Beijing, China) and sequenced for verification.

### 2.8. Yeast Two-Hybrid (Y2H) Assay

The *PpMYB1* gene was cloned into the pGADT7 vector, and the *PpbHLH1* gene was cloned into the pGBKT7 vector using homologous recombination. Primers used are listed in Table S1. The Y2H analysis followed Ye [46] and Suo [47]. After assessing the toxicity and self-activation of the pGBKT7-PpbHLH1 bait protein, pGBKT7-PpbHLH1 and pGBKT7-PpMYB1 were co-transformed into Y2H Gold yeast cells. These cells were then cultured sequentially in SD/-Trp-Leu + 10 mM 3-amino-1,2,4-triazole (3-AT) and SD/-Trp-Leu-His-Ade + 10 mM 3-AT + 30 mg/mL X-α-Gal media. Yeast growth was observed after 3 days at 30 °C. Negative controls included pGBKT7-Lam and pGADT7-T7, while positive controls were pGBKT7-53 and pGADT7-T7.

### 2.9. Transient Expression of PpMYB1 and PpbHLH1 in P. pulcherrima

*PpMYB1* and *PpbHLH1* were inserted into the pSuper1300-GFP vector using a TSINGKE TSV-S3 Trelief^®^ Seamless Cloning Kit (Tsingke, Beijing, China). The expression vectors generated were successfully transferred into the *Agrobacterium tumefaciens* strain EHA105. Flowers that were 3 to 5 days open were used for injections. Four types of engineered Agrobacterium tumefaciens were created: one with an empty vector, two with either 35S::*PpMYB1* or 35S::*PpbHLH1* individually, and one with an equal mixture of both. The flowers of *P. pulcherrima* were transiently transformed with each strain, and flower color alterations were monitored for 3 days post-transformation. Both the color changes and anthocyanin levels in the inoculated zones were documented. The experiment comprised three biological replicates to ensure reliability.

### 2.10. Stable Transformation of PpMYB1 and PpbHLH1 in Tobacco

Using homologous recombination, the *PpMYB1* gene was integrated into the stable transformation expression vector pGreen-C17. Approximately 5-week-old sterile tobacco plants were transformed and regenerated [35]. The recombinant expression vector, pGreen-C17-PpMYB1, was introduced into the *Agrobacterium tumefaciens* EHA105 strain. Leaf discs from tobacco plants were inoculated with *Agrobacterium tumefaciens* for 15 min, rinsed with sterile water, and placed on a shoot induction medium. After 3 days of incubation, adventitious shoots were selected on a medium containing 2 mg/L Basta. Once the shoots exceeded 3 cm in length, they were excised and transferred to a rooting medium. After root formation, the seedlings were transplanted into a lit growth chamber. Following molecular characterization via PCR, the seedlings were moved to a greenhouse, and flowers from the T0 generation of tobacco plants were collected for further analysis.

### 2.11. Yeast One-Hybrid (Y1H) Assay

The promoters of *PpF3H*, *PpDFR*, and *PpANS* were successfully cloned through the KX Genome Walking Kit (Zomanbio, Beijing, China), with the specific primer sequences provided in Table S1. To analyze cis-regulatory elements, the PlantCARE database (https://bioinformatics.psb.ugent.be/webtools/plantcare/html/ (accessed on 24 January 2025)) was utilized. For the Y1H assay, the promoter fragments were inserted into the pHis2 vector, while *PpMYB1* and *PpbHLH1* were cloned into the pGADT7-Rec2 vector. The oligonucleotide primers used for vector construction are listed in Table S1. The recombinant plasmids, pGADT7-Rec2 and pHis2, were subsequently introduced into the Y187 yeast strain. Various concentrations of 3-Amino-1,2,4-Triazole (3-AT) were incorporated into Synthetic Defined (SD)-Trp/-His medium, and yeast colony formation was monitored after 5 days of incubation at 30 °C to identify the optimal 3-AT concentration. Co-transformation of pGADT7-Rec2 and pHis2 into Y187 yeast strains was conducted, followed by incubation in SD/-Trp-Leu and SD/-Trp-Leu-His media for 3 days at 30 °C. The pGADT7-Rec2 vector was used as the control in this experiment.

### 2.12. Dual-Luciferase Assay

For the dual-luciferase assay, the promoters of *PpF3H*, *PpDFR*, and *PpANS* were cloned individually into the pGreenII0800-LUC vector, while *PpMYB1* and *PpbHLH1* were separately inserted into the pGreenII 62-SK vector using the infusion cloning technique (primer sequences are listed in Table S1). Six distinct vector combinations were co-infiltrated into tobacco leaves. At 48 h post-infiltration, leaf tissue samples were harvested, and the activities of Firefly Luciferase (LUC) and Renilla Luciferase (REN) were measured using a dual-luciferase reporter assay kit (Vazyme Biotech Co., Ltd., Nanjing, China), following the protocol described by Yin et al. [48]. Each experimental group included at least three independent biological replicates to ensure statistical robustness.

### 2.13. Statistical Analysis

The data are presented as mean values with standard deviations derived from three independent biological replicates. All data were analyzed using one-way ANOVA, followed by Duncan’s multiple range test, implemented in SPSS version 25.0 with a significance threshold set at *p* < 0.05. The resulting charts were constructed using Origin (version 9.8.0).

## 3. Results

### 3.1. The Flower Color of P. pulcherrima Is Related to the Content of Anthocyanin

**Figure 1:**
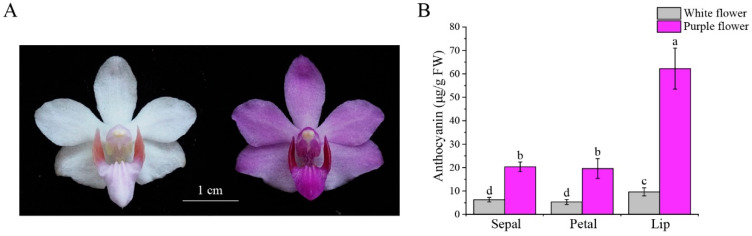
Phenotypic comparison and anthocyanin content of white and purple flower *P. pulcherrima* cultivars. (**A**) Photographs of white and purple flowers. (**B**) Comparison of total anthocyanin content of sepals, petals, and labellum between white and purple flower varieties. Letters above bars indicate significant differences at *p* < 0.05. The data show the means of three biological replicates ± SD.

To verify whether the color difference between purple and white flowers is due to variations in anthocyanin content, we analyzed the anthocyanin levels in the sepals, petals, and labellum of both purple and white flower varieties. We observed notable variations in anthocyanin content between the purple- and white-flowered varieties, with the purple variety exhibiting significantly higher anthocyanin levels in the sepals, petals, and labellum relative to the white variety (Figure 1A,B). Therefore, the measured anthocyanin levels demonstrate a strong association with the color traits of *P. pulcherrima*.

### 3.2. Transcriptome Data Analysis of White and Purple Flowers

To comprehend the molecular mechanisms behind color variation, we performed transcriptome sequencing analysis on white and purple flower tissues. A total of 40,354,672 to 52,466,880 raw reads were obtained. After quality control of the sequencing data, 40,041,258 to 52,027,300 reads were generated, with a Q30 base percentage exceeding 96.5% (Table S3). Using Trinity, all samples’ clean data were de novo assembled, and the assembly results were optimized and evaluated. After processing, the high-quality sequencing reads were assembled into 119,461 transcripts and 72,724 unigenes, with mean lengths of 1058 bp and 893 bp, respectively. The assembly achieved N50 metrics of 1771 bp for transcripts and 1592 bp for unigenes, as summarized in Table S4. The length distribution of these unigenes is shown in Table S5, where 34,619 (48%) unigenes exceed 500 bp in length. These single genes were used to identify putative genes related to flower color.

**Figure 2:**
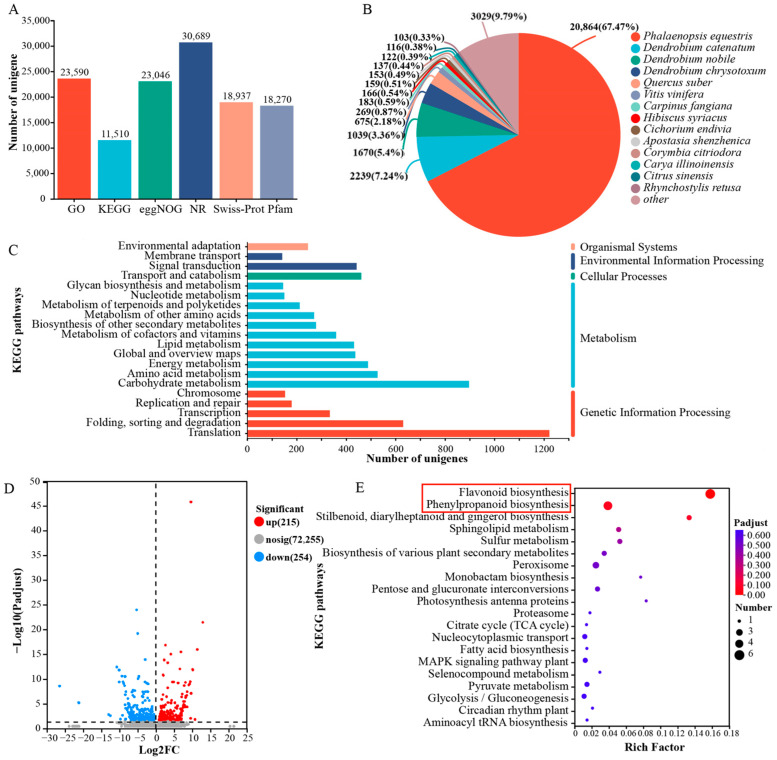
Summary of RNA sequencing results and unigene annotations. (**A**) Functional annotation of unigenes in six public databases. (**B**) Species distribution of unigenes in the NR database. (**C**) The unigenes’ functional classification in KEGG databases. (**D**) Volcano plot of DEGs between white and purple flowers. (**E**) KEGG enrichment of DEGs between white and purple flowers. The red boxes highlight key pathways potentially involved in pigment accumulation.

A sequence similarity analysis was performed using the BLASTX algorithm, comparing our protein sequences to those in multiple public repositories, with an *E*-value threshold established at 1 × 10^−10^. These genes underwent BLAST (https://www.ncbi.nlm.nih.gov/) searches in six public databases to infer their functional roles. The results revealed that 31,300 unigenes (43.04%) were successfully mapped to at least one of the six public databases (Figure 2A). In the NR database, 14 top-hit species derived from NR annotations were identified, with *P. equestris* exhibiting the highest similarity score to *Dendrobium officinale* (67.47%), followed by *Dendrobium catenatum* (7.24%), *Dendrobium nobile* (5.4%), and *Dendrobium chrysotoxum* (3.36%) (Figure 2B). KEGG pathway enrichment analysis revealed translation, carbohydrate metabolism, and folding, sorting, and degradation as the predominantly represented pathways (Figure 2C).

To identify genes associated with the pigmentation of *P. pulcherrima*’s purple petals, we identified 469 Differentially Expressed Genes (DEGs) (Figure 2D). KEGG enrichment analysis was conducted to determine their functions. Figure 2E displays the top 20 enriched KEGG pathways among the unigenes. Notably, pathways crucial for pigment accumulation, such as flavonoid and phenylpropanoid biosynthesis, were significantly enriched in the DEGs (Figure 2E).

### 3.3. Analysis of Anthocyanin Biosynthesis Pathway Genes

To better comprehend the pigmentation mechanisms in *P. pulcherrima* flowers, a thorough investigation of the genes linked to the color development pathway is crucial. Anthocyanin accumulation significantly influences the coloration of floral bracts, particularly in red flowers.

**Figure 3:**
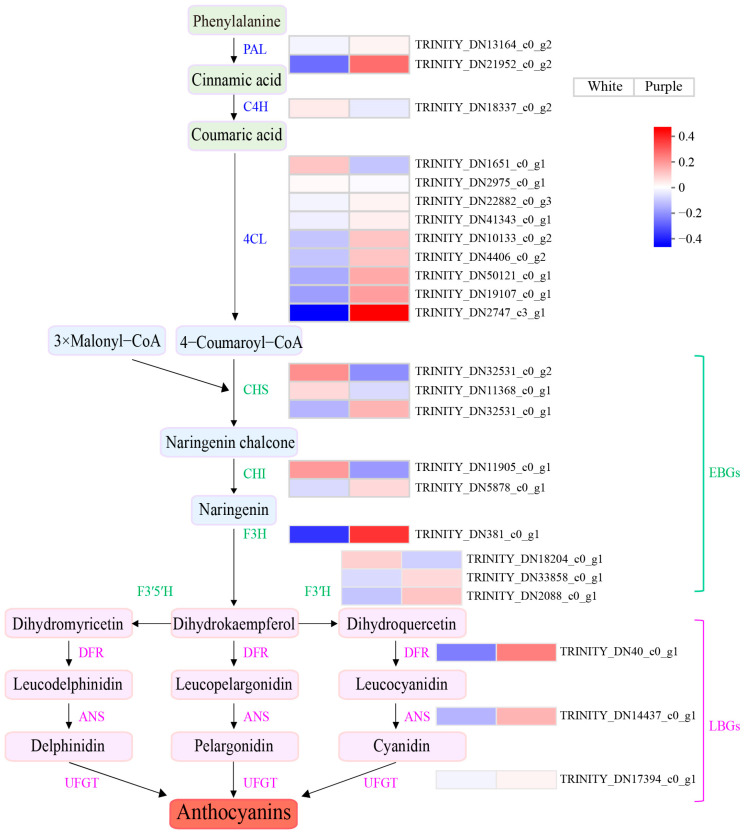
Heatmap generated of unigenes involved in anthocyanin biosynthesis. This visualization is constructed using the average expression levels of anthocyanin structural genes, calculated from TPM values obtained through RNA-seq analysis. Blue represents low expression levels, while red signifies high expression levels.

In this study, we have successfully identified the genes associated with the anthocyanin biosynthesis pathway; a total of 24 individual genes connected to anthocyanin production were identified, and an expression heatmap was generated using TPM values for visualization purposes (Figure 3). Nine EBGs involved in the anthocyanin pathway were identified: three *CHS*s, two *CHI*s, one *F3H*, and three *F3′H*s. *F3H* catalyzes the conversion of luteolin into the colorless dihydrokaempferol, which is the third critical rate-limiting enzyme in the anthocyanin pathway. The expression levels of *F3H* in purple flowers significantly differ from those in white flowers (Figure 3 and Table S6), and this analysis reveals that *F3H* plays a significant role in the development of purple pigmentation in *P. pulcherrima* flowers.

Furthermore, one *DFR*, one *ANS*, and one *UFGT* were identified as LBGs, and analysis of the expression heatmap demonstrates that these three downstream genes exhibit markedly elevated expression in purple flowers relative to white flowers. This suggests that their heightened activity within the anthocyanin biosynthesis pathway contributes to increased anthocyanin accumulation, which is responsible for the purple-red pigmentation observed in *P. pulcherrima* flowers (Figure 3 and Table S6).

### 3.4. Validate RNA-Seq Dataset Using qRT-PCR

**Figure 4:**
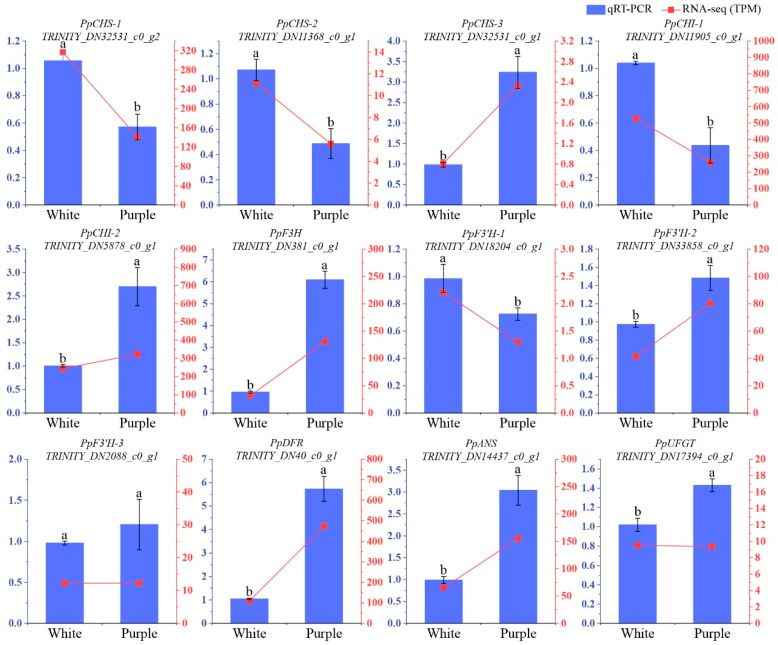
qRT-PCR validation of the expression profiles of selected unigenes related to anthocyanin biosynthesis. Twelve unigenes from this pathway were used to confirm the RNA-seq results. Expression levels were determined using the 2^−ΔΔCT^ method. The left *y*-axis shows the relative expression of the unigenes by qRT-PCR, while the right *y*-axis displays the TPM values from the RNA-seq data. Letters above bars indicate significant differences at *p* < 0.05. The data represent the mean of three biological replicates, with ±SD.

To ensure the accuracy of transcriptome sequencing results, we evaluated the expression profiles of 12 selected genes associated with anthocyanin biosynthesis in both white and purple flowers using qRT-PCR assays (Figure 4). We determined a significant correlation between the relative expression levels obtained from the RNA-Seq and qRT-PCR datasets, with a Pearson’s correlation coefficient of 0.9285 (Figure S1). Our RNA-seq results demonstrate strong agreement with qRT-PCR expression profiles, confirming the reliability of the transcriptomic data and supporting its applicability in downstream analyses.

### 3.5. Identification and Cloning of Key Transcription Factors

**Figure 5:**
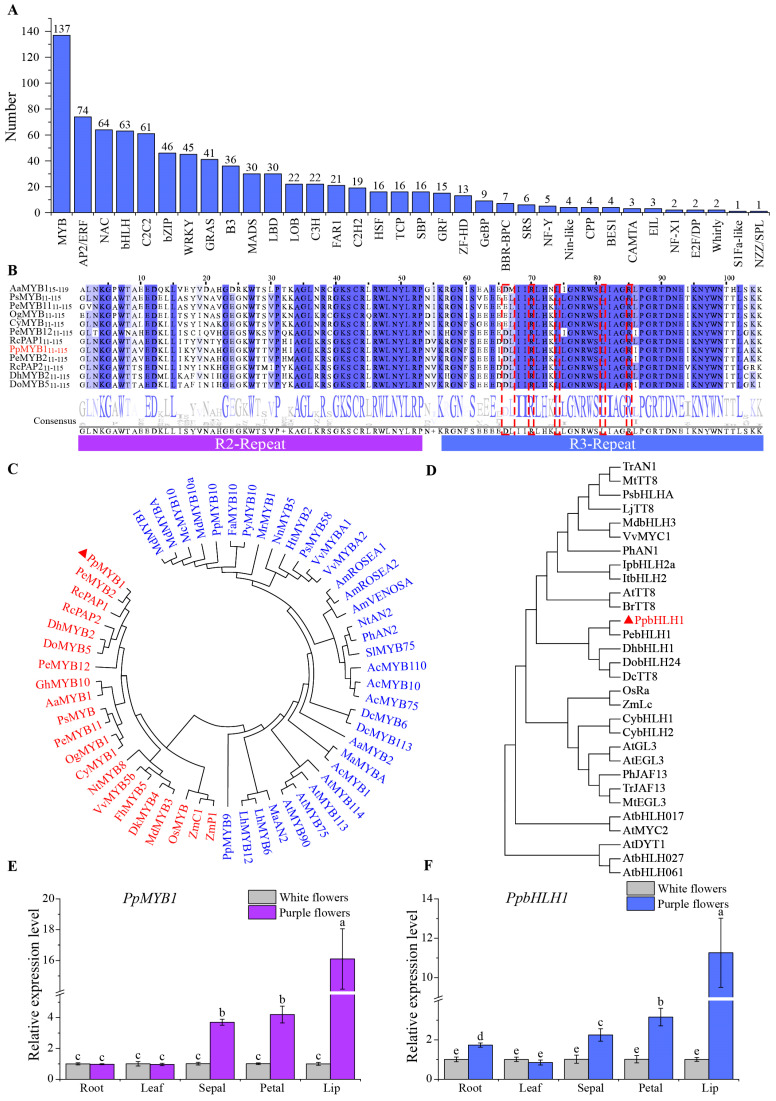
Phylogenetic analysis and relative expression level of *PpMYB1* and *PpbHLH1*. (**A**) Distribution of the number of transcription factor gene families in *P. pulcherrima*. (**B**) Comparison of the protein sequence of *PpMYB1* with its homologs from various species. The dashed red box indicates the interaction motif [D/E]LX_2_[R/K]X_3_LX_6_LX_3_R. (**C**,**D**) Phylogenetic analysis of PpMYB1 (**C**) and PpbHLH1 (**D**) compared to selected transcription factors from other plant species using the Neighbor-Joining method. (**E**) *PpMYB1* and (**F**) *PpbHLH1* expression levels in the root, leaf, sepal, petal, and lip of white and purple flower *P. pulcherrima* cultivars were measured using qRT-PCR. The data are means ± SD (n = 3). Letters indicate significant differences (*p* < 0.05, Duncan’s multiple range tests).

The regulation of flower pigmentation, particularly the biosynthesis of anthocyanins, heavily depends on the activity of transcription factors. Through RNA-seq annotation, we discovered and categorized 840 transcription factors distributed across 34 distinct families. The MYB family has the most members, with 137, while the bHLH family has 63 members (Figure 5A).

Additionally, we investigated the MYB transcription factors by integrating the expression profiles and sequence similarity assessments of 137 individual genes. We discovered that the levels of the gene *TRINITY_DN14368_c0_g1* were higher in purple varieties compared to white varieties, and it exhibits the highest similarity to reported anthocyanin activators in orchids (Figure 5B,C). Based on these findings, this gene was identified as a candidate MYB transcription factor involved in the regulation of anthocyanin biosynthesis and assigned the name PpMYB1 for further functional characterization. Complete cDNA sequences of *PpMYB1* were successfully amplified via PCR. The CDS of *PpMYB1* is 780 bp, encoding a polypeptide of 260 amino acids. Analysis of the amino acid sequence revealed that PpMYB1 contains a highly conserved R2R3 domain at its *N*-terminal end. Additionally, a protein interaction motif [D/E]LX_2_[R/K]X_3_LX_6_LX_3_R, facilitating interaction with bHLH transcription factors, was identified within the R3 domain of PpMYB1 (Figure 5B). The amino acid sequence of PpMYB1 exhibits 93% similarity to PeMYB2 from *P. equestris*, which is known to positively regulate anthocyanin accumulation. This high sequence homology implies that *PpMYB1* may share functional roles with *PeMYB2*, potentially contributing to anthocyanin biosynthesis (Figure 5B). Phylogenetic analysis also demonstrates that PpMYB1 clusters with other MYB transcription factors that promote anthocyanin biosynthesis, as indicated by red markers (Figure 5C).

Similarly, we identified a highly homologous *bHLH* single gene, *TRINITY_DN467_c1_g1*, through blast homology search. We named this gene *PpbHLH1*, which has a CDS of 1818 bp and encodes 606 amino acids. The phylogenetic analysis demonstrates that PpbHLH1 shares the closest homology with PebHLH1, which similarly relates to DhbHLH1 and DobHLH24 (Figure 5D).

The expression levels of *PpMYB1* and *PpbHLH1* were detected in the roots, leaves, sepals, petals, and lips of white- and purple-flowered plants, it can be seen that the relative expression levels in the various parts of *P. pulcherrima* flowers are notably higher than those in the white plants (Figure 5E,F), indicating that they significantly participate in the anthocyanin biosynthesis of the flowers. For example, the relative expression level of *PpMYB1* in the flower lip petals of the purple variety is 16.0 times that of the white variety (Figure 5E). The relative expression level of *PpbHLH1* is 11.3 times that of the white variety (Figure 5F). This suggests that the expression levels of *PpMYB1* and *PpbHLH1* are directly proportional to anthocyanin content, and their expression trends are consistent.

### 3.6. The Transient Overexpression of PpMYB1 and PpbHLH1 Enhances Anthocyanin Accumulation

**Figure 6:**
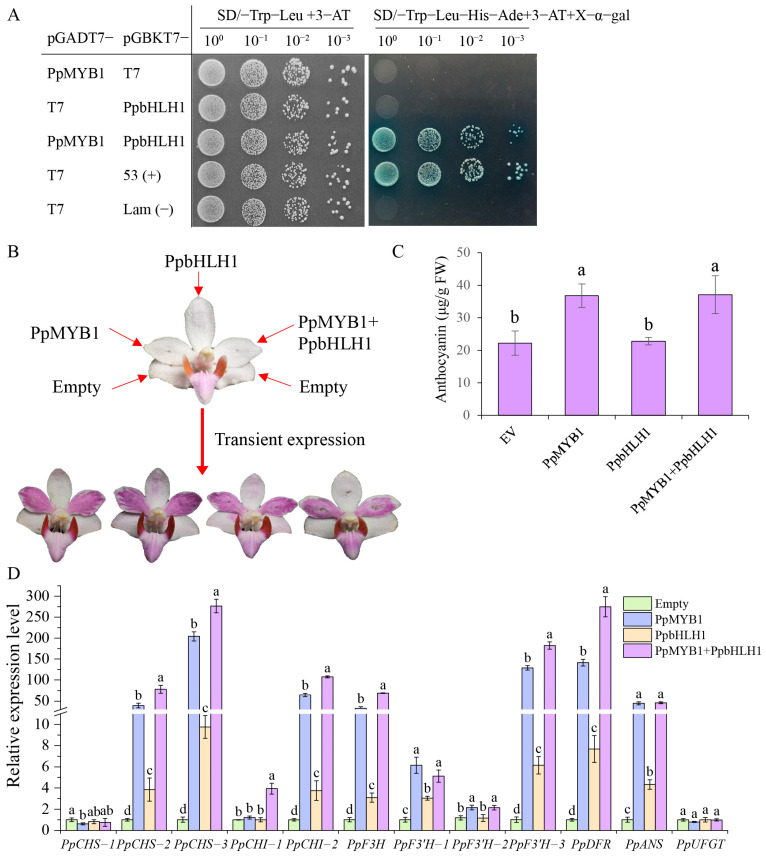
Detection of the interaction between *PpMYB1* and *PpbHLH1* and their transient overexpression in *P. pulcherrima* flowers. (**A**) The interaction between *PpMYB1* and *PpbHLH1* was analyzed using the Y2H system. The pGBKT7-Lam and pGADT7-T7 vectors served as negative controls, while the pGBKT7-53 and pGADT7-T7 vectors functioned as positive controls. (**B**) Photographs of the phenotypes from transient expression experiments of *PpMYB1* and *PpbHLH1* in *P. pulcherrima* flowers. (**C**) Measurement of total anthocyanin levels following the transient overexpression of *PpMYB1* and *PpbHLH1*. (**D**) Analysis of the expression levels of structural genes in the anthocyanin pathway following the transient overexpression of *PpMYB1* and *PpbHLH1*. The data are means ± SD (n = 3). Letters indicate significant differences (*p* < 0.05, Duncan’s multiple range tests).

To verify whether PpMYB1 and PpbHLH1 interact, we conducted a Y2H assay, which showed that both PpMYB1 and PpbHLH1 could grow on a two-deficient medium when combined. Separately, however, they failed to grow on a four-deficient medium, indicating that 3-AT had inhibited their autoactivation. When PpMYB1 and PpbHLH1 were introduced into yeast cells, their co-transformation enabled growth on a medium deficient in four components, with the addition of X-α-gal resulting in a blue color, a phenotype comparable to the positive control (Figure 6A). This suggests that *PpMYB1* and *PpbHLH1* can form heterodimers and interact with each other in vitro.

To further verify the functions of *PpMYB1* and *PpbHLH1* in the regulation of anthocyanin in *P. pulcherrima*, we injected Agrobacterium tumefaciens strain EHA105 carrying 35S::*PpMYB1* and 35S::*PpbHLH1* recombinant plasmids separately or simultaneously into *P. pulcherrima* flowers. We observed that petals injected with *PpMYB1* and the combination of *PpMYB1* and *PpbHLH1* turned distinctly purple (Figure 6A), and the total anthocyanin content measurements were consistent with the phenotypes (Figure 6B). Using qPCR, transcripts of genes related to anthocyanin biosynthesis were detected, showing significant upregulation for genes such as *PpCHS-2*, *PpCHS-3*, *PpCHI-2*, *PpF3H*, *PpF3′H-3*, *PpDFR*, and *PpANS* (Figure 6C). These findings suggest that overexpression of *PpMYB1* in white flower plant varieties facilitates anthocyanin biosynthesis, resulting in petals with purple-red pigmentation. In contrast, overexpression of *PpbHLH1* does not visibly alter petal color but partially enhances the expression of structural genes within the anthocyanin pathway. Interestingly, simultaneous overexpression of *PpMYB1* and *PpbHLH1* leads to elevated expression levels of structural genes in the anthocyanin pathway compared to *PpMYB1* alone, despite the lack of significant activation of the downstream gene *PpUFGT* (Figure 6D). In summary, transient expression experiments demonstrate that overexpression of *PpMYB1* and *PpbHLH1* promotes anthocyanin accumulation in Phalaenopsis flowers.

### 3.7. Stable Transformation of PpMYB1 and PpbHLH1 Genes in Tobacco

**Figure 7:**
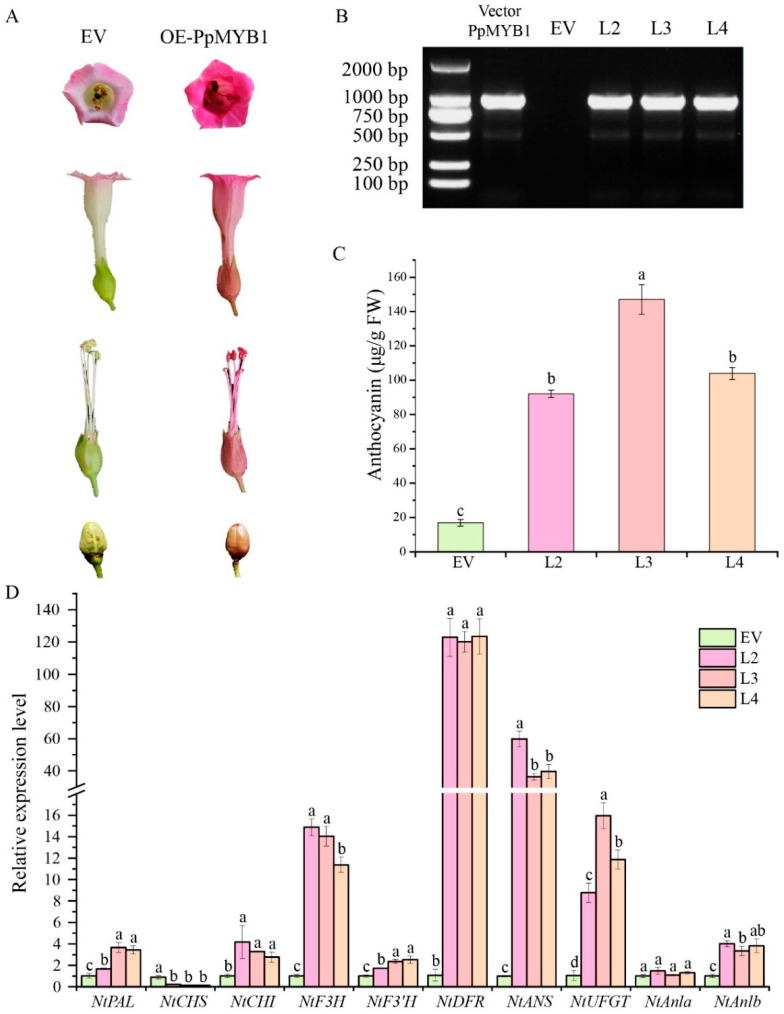
Analysis of transgenic tobacco flowers overexpressing PpMYB1 reveals insights into both phenotypic changes and molecular mechanisms. (**A**) Phenotypic analysis of flowers and fruits was conducted on *PpMYB1*-overexpressing lines and wild-type controls. EV refers to plants expressing an empty vector, while OE-*PpMYB1* denotes those with overproduction of *PpMYB1*. (**B**) Molecular identification of transgenic positive plants. (**C**) Detection of anthocyanin content in flowers of control and transgenic plants. (**D**) qRT-PCR analysis of flavonoid gene expression levels in tobacco flowers. Gene expression levels were normalized using tobacco tubulin. The data are means ± SD (n = 3). Letters indicate significant differences (*p* < 0.05, Duncan’s multiple range tests).

The experimental results are shown in Figure 7A. In transgenic tobacco flowers carrying overexpressed *PpMYB1*, the corolla color is significantly darker compared to control plants. In the *PpMYB1* overexpression lines, the reproductive organs exhibit a red phenotype, whereas the control plants maintain a green phenotype. Additionally, the seed coat color of the transgenic tobacco is redder (Figure 7A–C). Concurrently, the expression levels of EBGs related to anthocyanin biosynthesis, such as *NtCHI* and *NtF3H*, and LBGs like *NtDFR*, *NtANS*, and *NtUFGT* are significantly upregulated in the transgenic tobacco flowers. This upregulation may account for the observed phenotypic changes (Figure 7D). Furthermore, *Ntanlb* is also notably upregulated in the tobacco, indicating that the bHLH transcription factor in tobacco is activated. This suggests that the overexpression of transcription factor *PpMYB1* in tobacco promotes anthocyanin accumulation, resulting in purple-red flower coloration.

### 3.8. Transcriptional Regulation of Key Genes Involved in Anthocyanin Biosynthesis by PpMYB1 and PpbHLH1

**Figure 8:**
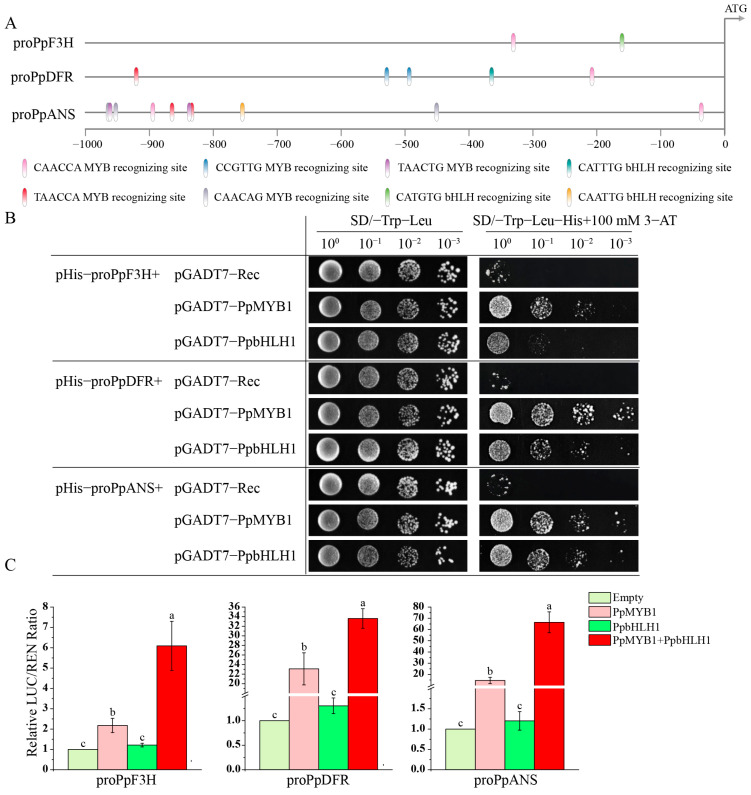
PpMYB1 and PpbHLH1 activate the promoters of *PpF3H*, *PpDFR*, and *PpANS*. (**A**) Distribution diagram of *PpF3H*, *PpDFR*, and *PpANS* promoter MYB- and bHLH-recognizing elements. pro: promoter. (**B**) Y1H assay detects the activation of downstream gene promoters by PpMYB1 and PpbHLH1. (**C**) The dual-luciferase reporter system is used to detect the activation of downstream gene promoters by PpMYB1 and PpbHLH1. The data are means ± SD (n = 3). Letters indicate significant differences (*p* < 0.05, Duncan’s multiple range tests).

To demonstrate that *PpMYB1* and *PpbHLH1* are capable of activating the promoters of downstream structural genes in the anthocyanin pathway and consequently controlling their transcriptional levels, we used chromosome walking technology to clone the promoter sequences of the LBGs *PpF3H*, *PpDFR*, and *PpANS* in the anthocyanin pathway. We then predicted *MYB* and *bHLH* recognition elements using PlantCARE. Among these, the promoter regions (−1000 bp) of *PpF3H*, *PpDFR*, and *PpANS* contain one, four, and nine MYB recognition elements, respectively, and one bHLH recognition element each (Figure 8A).

The promoter sequences of the four structural genes were independently transformed into Y187 competent yeast cells and subsequently cultured in SD selective medium (SD/-Trp-Leu) supplemented with 0–150 mM 3-AT. Yeast Y187 transformed with pHis-proPpF3H, -proPpDFR, and -proPpANS was inhibited by 3-AT at 100 mM. Co-expression of PpMYB1 and PpbHLH1 with pHis-proPpF3H, -proPpDFR, and -proPpANS in Y187 yeast showed that yeast co-expressing PpMYB1 with pHis-proPpF3H, -proPpDFR, and -proPpANS could grow in SD/-Trp-Leu-His medium containing 100 mM 3-AT, indicating strong interaction between PpMYB1 and the promoters of PpF3H, PpDFR, and PpANS. Similarly, PpbHLH1 also had strong interactions with the promoters of *PpDFR* and *PpANS* (Figure 8B).

Furthermore, to evaluate the transcriptional activation of PpMYB1 and PpbHLH1 on the promoters of *PpF3H*, *PpDFR*, and *PpANS*, a dual-luciferase assay was employed to assess their regulatory effects. PpMYB1 was found to significantly activate the promoters of *PpF3H*, *PpDFR*, and *PpANS*, whereas PpbHLH1 exhibited no activation effects on these promoters. However, when PpbHLH1 was co-expressed with PpMYB1, it significantly increased the activity of the promoters of *PpF3H*, *PpDFR*, and *PpANS*, particularly for the *PpANS* promoter, where the co-expression of PpMYB1 and PpbHLH1 resulted in an activity 4.7 times higher than that of PpMYB1 alone (Figure 8C).

**Figure 9:**
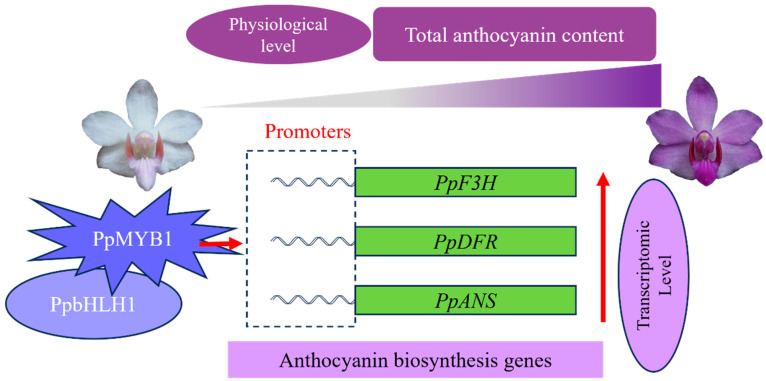
This model elucidates the regulatory process of the purple flowers in *P. pulcherrima*.

Finally, by integrating all experimental findings, we developed a theoretical framework that elucidates the regulatory mechanisms of *PpMYB1* and *PpbHLH1* in the anthocyanin biosynthesis pathway of *P. pulcherrima* (Figure 9).

## 4. Discussion

*P. pulcherrima* is an ornamental plant with diverse flower colors and a flowering period that does not overlap with that of *Phalaenopsis* [49]. It has strong disease and stress resistance [50], thus attracting widespread attention from breeders for hybridization with orchid plants such as *Phalaenopsis* [51]. Despite its appeal, the molecular mechanism behind anthocyanin biosynthesis in *P. pulcherrima* remains incompletely understood [52]. Furthermore, the lack of a reference genome significantly limits research efforts in unraveling the molecular mechanisms underlying key traits in this plant [53]. RNA sequencing offers an efficient and economical approach to analyze gene expression and identify critical genes in plants without a reference genome, across different developmental stages and physiological conditions [54,55]. Li et al. [56] performed a transcriptomic comparison between red and white Primula vulgaris petals, identifying the key genetic factors underlying anthocyanin pigmentation and investigating the associated genetic regulation and biochemical pathways. Consistent with this, we also used RNA transcriptome sequencing to analyze the transcription of white and purple flowers of *P. pulcherrima*. Out of 31,300 unigenes (43.04%), annotations were successfully made to at least one of six public databases. In the NR database, the highest match for annotation was with *P. equestris* (67.47%). KEGG enrichment analysis was performed on the 469 identified differentially expressed genes. It was found that key pathways involved in pigment accumulation, flavonoid biosynthesis, and phenylpropanoid biosynthesis were significantly enriched among these genes (Figure 2). Unfortunately, our study did not involve individual RNA sequencing of sepals, petals, and lip across distinct developmental stages, which prevents us from analyzing the temporal variations in gene expression or identifying organ-specifically expressed genes in different flower organs.

Transient expression and stable expression are important methods for verifying gene function [57]. Transient expression refers to the process of introducing exogenous genes into cells and obtaining gene expression products within a short period of time. This method is suitable for rapid screening and preliminary verification of gene function [58]. Stable expression, on the other hand, involves integrating exogenous genes into the cell genome, allowing them to be continuously and stably expressed in the cell [59]. Compared with transient expression, stable expression enables longer-term observation of gene function and effects [60], providing a powerful tool for further revealing the mechanism of gene action. For example, the seeds of transgenic *Arabidopsis* plants overexpressing *PSC1* genes from peanuts were observed to be a deeper purple compared with the wild type. Similarly, peanut callus overexpressing *PSC1* exhibited a deeper purple color and higher anthocyanin content compared with the wild-type callus [61]. Most studies on orchids use *P*. *aphrodite* flower petals as materials for transient expression. Transient overexpression of *RcPAP1* and *RcPAP2* in the perianths of *Cattleya hybrid* ‘KOVA’ achieved substantial cyanin accumulation, resulting in purple-red or pink pigmentation [62]. In this study, we optimized the transient expression protocol and applied it for the first time to the flowers of *P. pulcherrima*. As shown in Figure 6, injecting *PpMYB1* alone or co-injecting it with *PpbHLH1* resulted in a noticeable purple discoloration of the white petals, accompanied by a strong induction of genes associated with the anthocyanin biosynthesis pathway. This represents the first attempt to perform transient expression in *P. pulcherrima* flowers. This approach could be adapted and refined for other flowering plants to facilitate rapid screening and characterization of candidate genes. Although *Phalaenopsis* has a relatively well-established stable genetic transformation system, the long time required for plants to flower (2–3 years) makes it impractical for quick phenotypic analysis [63,64]. Similarly, establishing a stable transformation system for *P. pulcherrima* has proven challenging. Wang et al. [65] introduced a method involving ovary injection for genetic transformation, but its low efficiency and the extended waiting period for flowering (3–4 years) render it unsuitable for rapid phenotypic evaluation. Therefore, we utilized the model plant tobacco for stable expression. When *PpMYB1* was stably transferred into tobacco, the transgenic plants exhibited significantly higher anthocyanin content and darker purple pigmentation in their corolla, stigma, and pods compared to control plants. In summary, transient and stable expression methods serve as complementary approaches, providing a valuable tool for validating gene functions in plant systems.

In this study, we employed the chromosome walking technique to isolate upstream regulatory regions of key structural genes, including *PpF3H*, *PpDFR*, and *PpANS*, which are integral to anthocyanin biosynthesis. We identified the presence of MYB and bHLH transcription factor binding motifs within these promoter sequences. Our experimental findings demonstrated that PpMYB1 interacts directly with and significantly enhances the transcriptional activity of these genes. In contrast, PpbHLH1 alone showed minimal activation, yet its co-expression with PpMYB1 resulted in a pronounced synergistic effect. Notably, this co-expression elevated the promoter activity of *PpANS* by 4.7-fold compared to PpMYB1 alone (Figure 8). The inability of PpbHLH1 to activate promoters independently suggests a dependency on auxiliary factors, such as PpMYB1 or WD40 repeat proteins. Interestingly, *GhMYB1a* in *Gerbera hybrida* functions as a homodimer rather than interacting with bHLH co-factors [66]. This cooperative interaction could be analogous to the mechanism observed in *DoMYB5* and *DobHLH24*, where MYB and bHLH transcription factors assemble a dyad complex that interacts with MYB and bHLH elements within the promoter regions to enhance transcription initiation more efficiently [67]. The differential abundance of MYB and bHLH motifs in various gene promoters, particularly the nine MYB elements in *proPpANS*, indicates that *PpANS* expression is subject to more rigorous transcriptional regulation or that its promoter displays heightened sensitivity to transcription factors. These findings underscore the pivotal roles of PpMYB1 and PpbHLH1 in anthocyanin biosynthesis regulation, with *PpMYB1* emerging as a central regulatory component. The results contribute novel insights into the molecular regulation of anthocyanin synthesis and offer potential avenues for metabolic engineering applications in plant genetics.

As in most reports on flower color studies in orchidaceous plants, our research has also not identified any potential WD40 proteins that promote the accumulation of anthocyanins. Based on this, we speculate that WD40 proteins may not be essential for the interaction between MYB and bHLH proteins or for the regulation of anthocyanin biosynthesis, and their roles may vary depending on the plant species, exhibiting both conserved and species-specific functions.

PpMYB1 functions as a key transcriptional activator in anthocyanin biosynthesis and has been demonstrated to enhance anthocyanin content in multiple plant species through overexpression or gene editing strategies. Furthermore, the cooperative interaction between PpbHLH1 and PpMYB1 suggests that simultaneous introduction of these transcription factors in genetic manipulation could more effectively drive the expression of anthocyanin biosynthesis-related genes, leading to an increased anthocyanin production. Future research should aim to elucidate the interaction networks of additional regulatory factors influencing pigment synthesis to achieve a comprehensive understanding of the anthocyanin biosynthesis regulation mechanisms.

## 5. Conclusions

This research elucidated the regulatory process of flower color in *Phalaenopsis* by comparing the physiological and transcriptomic levels of white and purple flowers. We observed that the formation of purple flowers is mainly due to an increase in anthocyanin content. The RNA-seq analysis revealed a total of 469 DEGs, comprising 215 upregulated and 254 downregulated transcripts. Notably, a marked enrichment of genes involved in the biosynthesis of flavonoids and phenylpropanoids was observed, suggesting their critical roles in the regulatory network under investigation. Further analysis revealed that key transcription factors *PpMYB1* and *PpbHLH1* constitute heterodimers, which trigger robust expression of critical structural genes *PpF3H*, *PpDFR*, and *PpANS* in the anthocyanin biosynthesis pathway. This interaction leads to the development of purple flowers. In summary, this study is the first to elucidate the regulatory mechanism underlying purple flower formation in *P. pulcherrima*, marking the first scientific report on the flower coloration of this species. We have demonstrated the critical roles of *PpMYB1* and *PpbHLH1* in controlling anthocyanin biosynthesis through validations in both the plant itself and a tobacco stably transformed system. Specifically, our findings identify *PpMYB1* as a primary transcriptional regulator and highlight the synergistic interaction between PpbHLH1 and PpMYB2 in this process. These insights not only advance our understanding of the molecular mechanisms governing anthocyanin synthesis but also hold practical significance for manipulating anthocyanin biosynthesis in plant genetic engineering applications.

## Data Availability

Data may be found within the article or Supplementary Materials. Raw reads were deposited in the NCBI database under BioProject number PRJNA1255783. Sequence data from this article can be found in the GenBank data libraries under accession numbers: *PpMYB1* (PV451623) and *PpbHLH1* (PV451623).

## Supplementary Materials

The following supporting information can be downloaded at: https://www.mdpi.com/article/10.3390/biom15070906/s1, Table S1. Primers used in the present study; Table S2. The accession numbers of MYB and bHLH proteins. Table S3. Summary of Illumina sequencing data; Table S4. Evaluation of sequencing assembly data; Table S5: Distribution of Unigene length; Table S6: Transcripts Per Million mapped reads (TPM) values of anthocyanin biosynthetic genes. Figure S1. Correlation analysis based on RNA-seq data and real-time PCR.

## Abbreviations

GO: gene ontology; KEGG: Kyoto Encyclopedia of Genes and Genomes; COG: clusters of orthologous groups of proteins; NR: non-redundant protein sequence database; Swiss-Prot: a manually annotated, non-redundant protein sequence database; qRT-PCR: quantitative real-time polymerase chain reaction; PAL: phenylalanine ammonia-lyase; C4H: trans-cinnamate 4-monooxygenase; 4CL: 4-coumarate-CoA ligase 2; CHS: chalcone synthase; CHI: chalcone isomerase; F3H: flavanone 3-hydroxylase; F3′H: flavonoid 3-hydroxylase; F3′5′H: flavonoid 3′,5′-hydroxylase; DFR: dihydrofavonol 4-reductase; ANS: anthocyanidin synthase; UFGT: UDP-flavonoid glucosyl transferase; EBGs: early biosynthesis genes; LBGs: late biosynthesis genes.
