# Supplementary figures and images for: Transcriptome Analysis Reveals PpMYB1 and PpbHLH1 Promote Anthocyanin Accumulation in Phalaenopsis pulcherrima Flowers
Source: Biomolecules. 2025 Jun 20;15(7):906. doi: 10.3390/biom15070906 (PMC12292641; doi:10.3390/biom15070906)

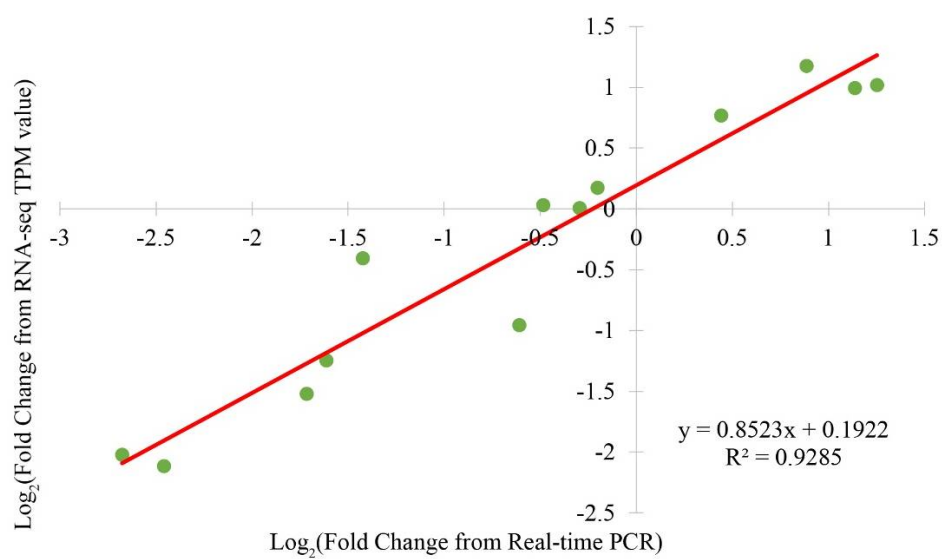

**Figure S1.** Correlation analysis based on RNA-seq data and real-time PCR.

Supplement: Supplementary file 1 [file biomolecules-15-00906-s001.zip › biomolecules-3651021-Figure S1.pdf]
